# Recent Advances on Biological Activities and Structural Modifications of Dehydroabietic Acid

**DOI:** 10.3390/toxins14090632

**Published:** 2022-09-12

**Authors:** Meng Hao, Jianwei Xu, Houpeng Wen, Jiawei Du, Shaoyong Zhang, Min Lv, Hui Xu

**Affiliations:** 1 College of Plant Protection, Northwest A&F University, Yangling 712100, China; 2 Key Laboratory of Vector Biology and Pathogen Control of Zhejiang Province, College of Life Science, Huzhou University, Huzhou 313000, China

**Keywords:** dehydroabietic acid, bioactivity, structural modification, total synthesis, structure-activity relationship

## Abstract

Dehydroabietic acid is a tricyclic diterpenoid resin acid isolated from rosin. Dehydroabietic acid and its derivatives showed lots of medical and agricultural bioactivities, such as anticancer, antibacterial, antiviral, antiulcer, insecticidal, and herbicidal activities. This review summarized the research advances on the structural modification and total synthesis of dehydroabietic acid and its derivatives from 2015 to 2021, and analyzed the biotransformation and structure-activity relationships in order to provide a reference for the development and utilization of dehydroabietic acid and its derivatives as drugs and pesticides.

## 1. Introduction

**Figure 1:**
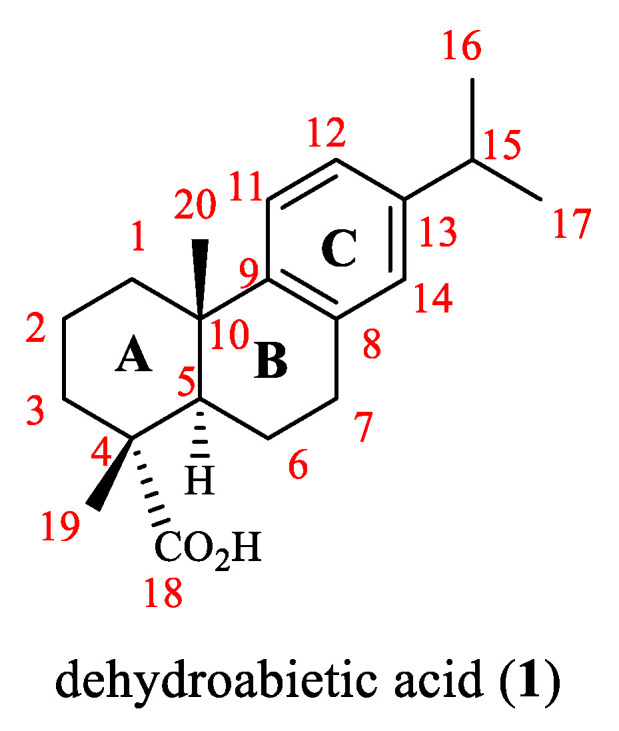
Chemical structure of dehydroabietic acid (**1**).

Terpenoids containing a variety of biological activities, are the promising lead structures for the development of drugs and pesticides [1,2,3,4,5,6]. Rosin is an important natural renewable resource in pine trees [7,8]. Dehydroabietic acid (**1**, Figure 1), a tricyclic diterpenoid, is a natural resin acid isolated from rosin and shows a wide range of biological activities [9]. The content of dehydroabietic acid in rosin was low, and it was not suitable for direct extraction of dehydroabietic acid. Storage-stable dehydroabietic acid can be purified by disproportionating abietic acid or rosin. In the industry, disproportionated rosin was obtained by disproportionation reaction of rosin as raw material, and then dehydroabietic acid can be obtained by organic amine salt and solvent recrystallization methods. Therefore, the research focus of purification was mainly on the preparation of disproportionated rosin, and the most effective catalyst for the disproportionation process of rosin was the expensive Pd/C catalyst, so it was very important to seek an efficient and economical purification condition [10]. In addition, dehydroabietic acid can also be isolated from two cyanobacteria strains [11].

The molecular skeleton of dehydroabietic acid contains one carboxyl group, one aromatic ring, and two alicyclic rings, with a total of twenty carbons. Dehydroabietic acid and its derivatives showed a variety of biological activities such as antiviral [12], antitumor [13], wound-healing [14], antiulcer [15], gastroprotective [16], anxiolytic [17], herbicidal [18] and antibacterial properties [19]. Furthermore, Xie et al. found that dehydroabietic acid had insecticidal activity against *Peridroma saucia* (Lepidoptera: Noctuidae) [20]. It demonstrated that larch sawfly exposure to dehydroabietic acid resulted in reduced feeding and slowed growth [21]. Dehydroabietic acid acted as an antagonist of insect juvenile hormone, interfering with the endocrine regulation of insects [22], and its derivatives also had an attracting effect on *Spodoptera litura* [23]. Recently, Xin et al. reported that novel multifunctional nanomedicines assembled from chitosan oligosaccharide-melanin complexes and dehydroabietic acid hexamers can achieve efficient and precise treatment of tumors [24]. Huang et al. found that biomass-based carbon dots prepared from dehydroabietic acid by hydrothermal reaction not only can sensitively and selectively detect heavy metal ions, but also can be used for cell imaging with low cytotoxicity [25]. However, while its biological activities were diverse, it had certain toxic and side effects. It reported that resin acids may be toxic to fish [26,27]. Based on the above studies, with the characteristics of good stability (due to the aromatic ring) and wide biological activities, dehydroabietic acid was a candidate of interest in the fields of medicine and agriculture. Moreover, to solve the disadvantages of its toxic and side effects, it was necessary to develop new dehydroabietic acid derivatives with high bioactivity and low toxicity. Therefore, this review summarized the structural modifications and biological activities of dehydroabietic acid and its derivatives from 2015 to 2021.

## 2. Bioactivities of Dehydroabietic Acid and Its Derivatives

### 2.1. Antitumor Activity

**Figure 2:**
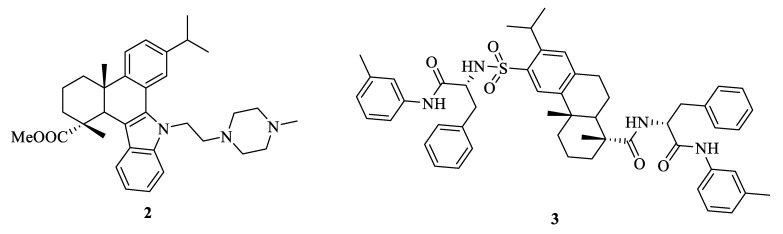
Chemical structures of compounds **2** and **3**.

Dehydroabietic acid derivatives exhibited anti-cancer activity through various mechanisms including inhibiting tumor cell migration and inducing tumor apoptosis [7]. Lee et al. found that dehydroabietic acid induced apoptosis of human lung cells by inducing the division of caspase-3 and PARP in these cells and interfering with mitochondria [28]. Moreover, Luo et al. suggested that a derivative of dehydroabietic acid (**2**, Figure 2), maybe a therapeutic drug for gastric cancer because it induced damage to cell membranes and organelles, and ultimately led to apoptosis of gastric cancer cells [29]. Through flow cytometry and cell cycle analysis, it was found that compound **3** (Figure 2) can induce HepG2 cell apoptosis and block the HepG2 cell line in the G1 phase to exert anti-cancer effects [30]. Dehydroabietic oxime was a possible method for the treatment of pancreatic cancer and its related inflammation. It can up-regulate the level of p27, and it can also down-regulate the expression of cyclin D1, thereby preventing the growth of pancreatic cancer cells in the G1 phase [31]. Recently, some dehydroabietic acid derivatives were potential drugs targeting the kinase domain of EGFR, which showed anticancer activity against HepG2 cancer cell lines [32].

### 2.2. Anti-Inflammatory Activity

Kim et al. evaluated the anti-inflammatory effects of dehydroabietic acid and found that not only the production of nitric oxide (NO) in the macrophage cell line was reduced under the action of dehydroabietic acid, but also the expression of inflammatory genes was descended. In addition, through the treatment of dehydroabietic acid, both the activity of kinases in the NF-κB cascade and TAK1 (transforming growth factor β-activated kinase 1) in the AP-1 cascade was inhibited [33]. A study conducted by Kang et al. proved that the treatment of dehydroabietic acid was an effective way to ameliorate inflammatory changes related to obesity-related diabetes. It demonstrated that dehydroabietic acid was an activator of PPARα and PPARγ and can inhibit the production of MCP-1, TNF-α, and NO (pro-inflammatory mediators) [34].

### 2.3. Antibacterial and Antifungal Activities

**Figure 3:**
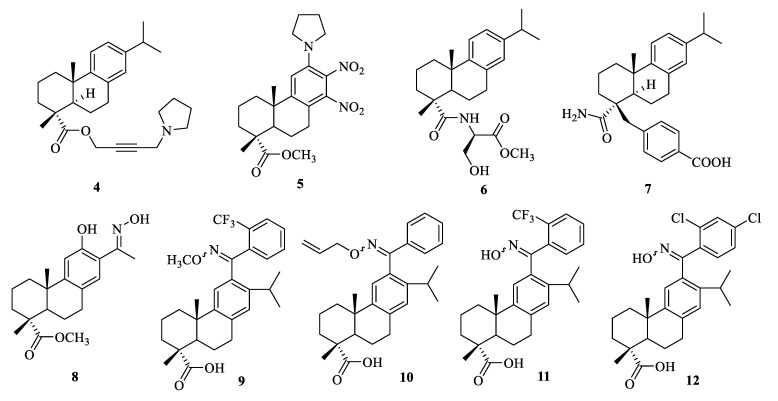
Chemical structures of compounds **4**−**12**.

Experiments by Tretyakova et al. proved that pyrrolidine-containing dehydroabietic acid acetylene derivatives (**4**, Figure 3) showed growth inhibitory effects on the fungi *Candida albicans* and *Cryptococcus neoformans*, and had low hemolytic capacity [35]. In addition, Chen et al. reported that the derivative of dehydroabietic acid (**5**, Figure 3) exhibited excellent antibacterial activity against Gram-positive bacteria such as *Bacillus subtilis* and *Staphylococcus aureus* with the MIC (minimum inhibitory concentration) value of 4 and 2 μg/mL, respectively [36]. Dehydroabietic acid-containing serine derivatives had excellent antibacterial activity against Gram-positive bacteria. For example, the MIC_90_ values of compound **6** (Figure 3) against methicillin resistant *S. aureus*, *Staphylococcus epidermidis,* and *Streptococcus mitis* were 8 μg/mL [19]. Furthermore, through the broth micro-dilution method, it was found that dehydroabietic acid showed antibacterial activity against four kinds of *Streptococcus mutans* (including *S. mutans* ATCC 12175, *S. mutans* NRPC 801, *S. mutans* NRPC 804, and *S. mutans* DMST 18777) [37]. Hassan et al. used dehydroabietic acid derivatives to prepare antibacterial nanocellulose membranes to solve the inevitable toxicity of silver and cationic antimicrobial agents. By simulating the physiological environment of chronic wounds, they found that it prevented the colonization of bacteria on the surface [38]. It was revealed that the dehydroabietic acid analog (**7**, Figure 3) exhibited antimicrobial activity against methicillin resistant *S. aureus* (MIC: 32 μg/mL) [39]. It is urgent to develop new antibacterial agents against methicillin-resistant and methicillin-sensitive *S. aureus* infections (MRSA and MSSA). Compound **8** (Figure 3) had effective inhibitory activity against the above-mentioned bacteria, and its MIC values were from 3.9 to 15.6 μg/mL. Moreover, it had no cytotoxicity and hemolytic activity in mammalian cells [40]. In addition, Liu et al. elucidated that some 12-oxime and *O*-oxime ether derivatives of dehydroabietic acid had strong antistaphylococcal activity. Compound **9** showed high inhibitory activity against *S. aureus* Newman with MIC value of 0.39–0.78 μg/mL, while compounds **10**−**12** had MIC values of 1.25–3.13 μg/mL for multidrug-resistant *S. aureus.* [41]. Additionally, some MIC values of dehydroabietic acid derivatives were listed in Table 1.

### 2.4. Insecticidal Activity

**Figure 4:**
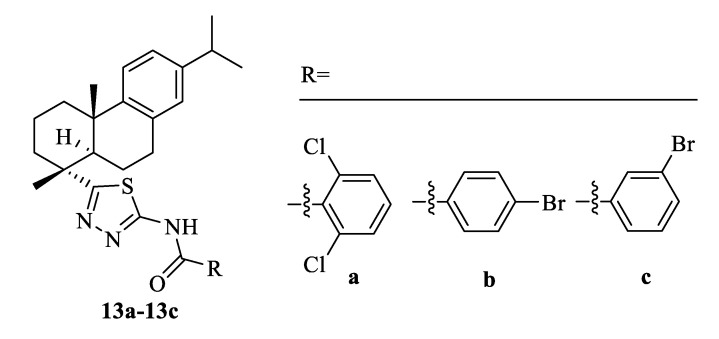
Chemical structures of compounds **13a**−**13c**.

Dehydroabietic acid displayed antifeedant activity against Indian meal moth *Plodia interpunctella*, but had no obvious effect on the growth of larvae [22]. Additionally, dehydroabietic acid had certain insecticidal activity against *Aedes aegypti* larvae, with a lethality rate of 65% at 10 ppm [42]. Gao et al. found that dehydroabietic acid amide derivatives containing the thiadiazole fragment (**13a**−**13c**, Figure 4) exhibited remarkable insecticidal activities against diamondback moth (*Plutella xylostella*) with the LC_50_ of 0.222–0.224 μg/mL [43]. Dehydroabietic acid had good antifeedant activity and a certain toxic effect on *Mythimna separata* larvae [44]. Moreover, the mountain pine beetle was often affected by the chemical defense of host oleoresin secretions which contained dehydroabietic acid [45].

### 2.5. Antiprotozoal Activity

Dehydroabietic acid not only inhibited the proliferation of the promastigote form, but also induced the production and output of ROS (reactive oxygen species) and downregulated Nrf2 (nuclear factor erythroid 2-related factor 2) to exert its anti-Leishmania activity, mainly due to its antioxidant properties [46]. Pertino et al. reported that dehydroabietic acid had certain antiprotozoal activity against *Trypanosoma cruzi*, *Leishmaniabraziliensis*, and *Leishmania infantum* [47]. Moreover, the dehydroabietic acid derivative containing amino acid structure was a new and effective antiprotozoal drug with good selectivity to *Leishmania donovani* and *T. cruzi* [48].

### 2.6. Other Activities

**Figure 5:**
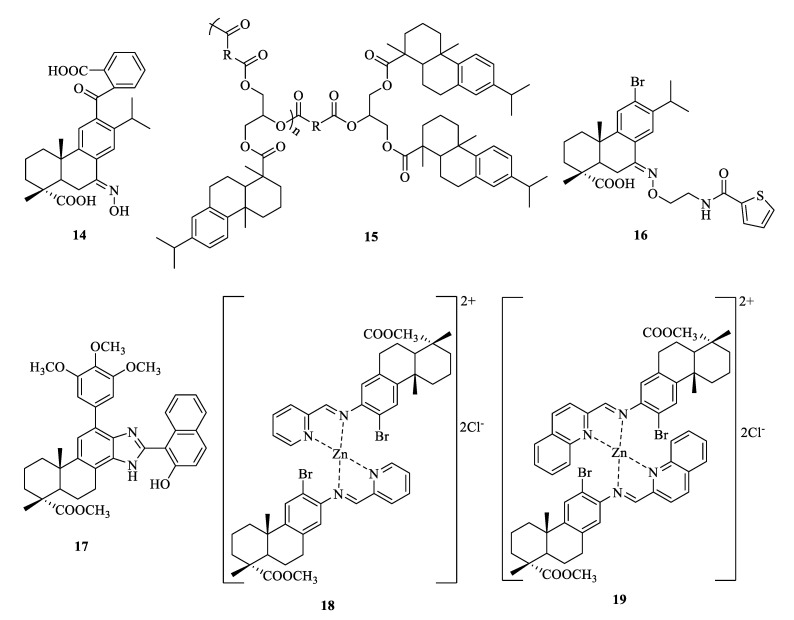
Chemical structures of compounds **14**−**19**.

The treatment of dehydroabietic acid alleviated insulin resistance and liver steatosis caused by a high-fat diet in mice as a dual agonist of PPAR-α/γ [49]. Moreover, the anti-obesity effect of dehydroabietic acid may be exerted by improving the levels of plasma glucose and insulin via PPARα/γ-dependent pathways [34,50,51]. Dehydroabietic acid reduced hypertrophy by activating the Keap1/Nrf2-ARE signaling pathway, increasing the expression of the ferroptosis suppressor protein 1 (FSP1) gene, and inhibiting the accumulation of ROS to ameliorate non-alcoholic fatty liver disease [52]. Dehydroabietic acid inhibited angiotensin converting enzyme in human umbilical vein endothelial cells and induced p-Akt, which could reduce the systolic blood pressure of spontaneously hypertensive rats, thereby exerting an anti-hypertensive effect [53]. Dehydroabietic acid as an anti-aging agent could directly bind to SIRT1 protein and activated SIRT1 [54]. Park et al. suggested that dehydroabietic acid protected and regenerated the collagen fibers in the skin irradiated by ultraviolet B [55]. In addition, through *in vivo* and *in vitro* experiments, it was found that the assembled dehydroabietic acid derivative (**14**, Figure 5)/ZnAlTi-LDH (layered double hydroxide) composite material can promote wound healing, kill Gram-negative and positive bacteria infecting the wound, and block ultraviolet rays to protect the skin [56]. Since dehydroabietic acid can promote osteogenic differentiation, it may be a new idea for the treatment of osteoporosis in the elderly [57]. Through *in vitro* experimental studies, Nachar et al. found that dehydroabietic acid may be an effective drug for the treatment of type 2 diabetes. It not only reduced the activity of G6Pase to descend glucose production but also stimulated GS activity to ascend glucose storage [58]. Additionally, a dehydroabietic acid polymer (**15**, Figure 5) can be used as a toner for xerography [59]. BK channels, K^+^ channels activated by large-conductance calcium, had vital physiological functions such as the release of neurotransmitters. A thiophene derivative of dehydroabietic acid (**16**, Figure 5) showed BK channel opening activity [60,61]. A dehydroabietic acid derivative (**17**, Figure 5) can be used as fluorescent probes and can sensitively detect Fe^3+^ and Hg^2+^ ions in cells [62]. Moreover, dehydroabietic acid derivatives (**18** and **19**, Figure 5) as natural resources provided a new method for the synthesis of fluorescent materials [63].

## 3. Structural Modification of Dehydroabietic Acid and Its Derivatives

### 3.1. Structural Modification at C-18 of Dehydroabietic Acid

**Scheme 1:**
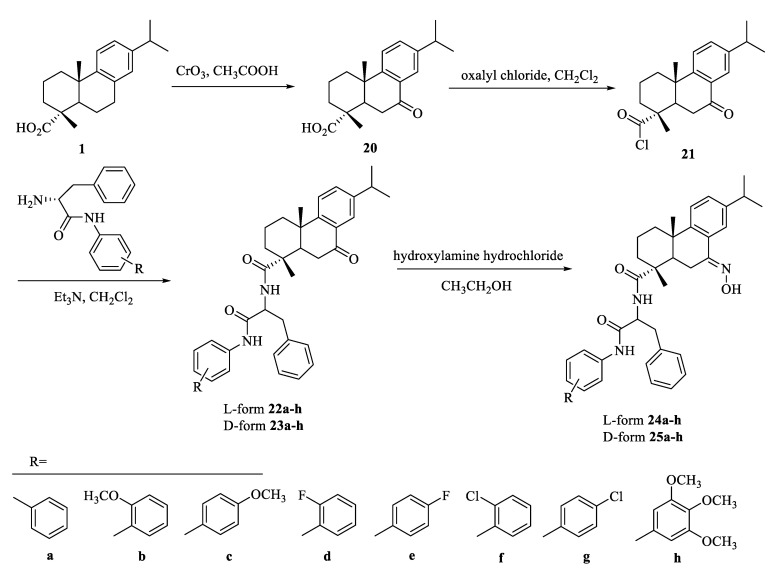
Synthesis of compounds **22**(**a**-**h**)−**25**(**a**-**h**).

As depicted in Scheme 1, Huang et al. synthesized a series of chiral dipeptide derivatives (**22**(**a**-**h**)−**25**(**a**-**h**)) of dehydroabietic acid and tested their anticancer activities by the MTT method against HeLa, NCI-H460, and MGC-803 tumor cell lines. Among them, compound **22f** had the strongest inhibitory effect on the HeLa cancer cell line, with an IC_50_ value of 7.76 ± 0.98 μM, and induced cell apoptosis through the mitochondrial pathway [64]. 

**Scheme 2:**
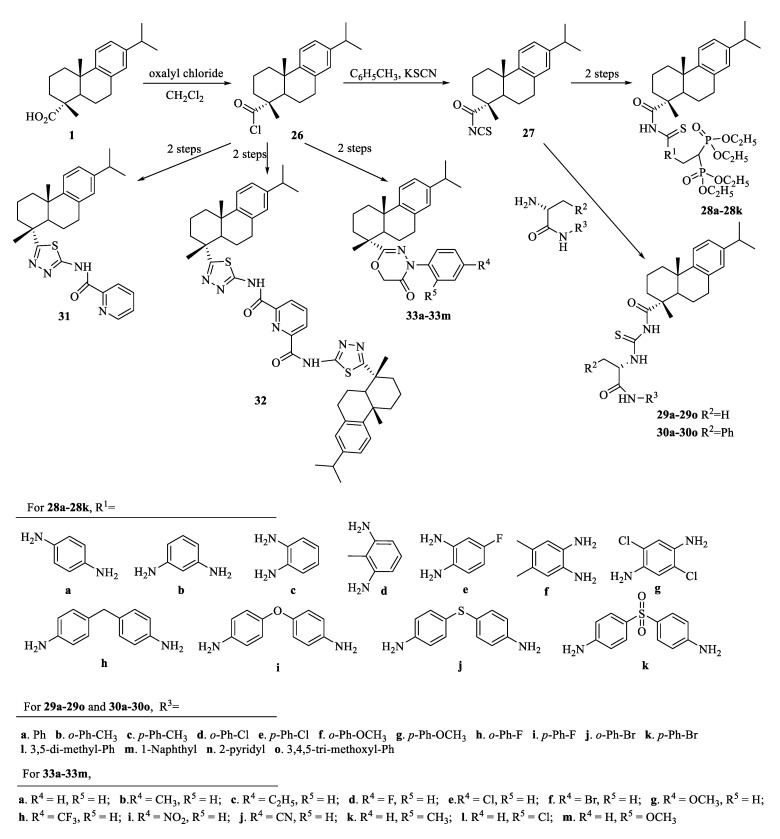
Synthesis of compounds **28a**−**28k**, **29a**−**29o**, **30a**−**30o**, **31**, **32** and **33a**−**33m**.

Subsequently, compounds containing thiourea and bisphosphonates (**28a**−**28k**) were synthesized as effective antitumor agents (Scheme 2). Compound **28e** showed the best anticancer activity against the SK-OV-3 cell line with an IC_50_ value of 1.79 ± 0.43 μM, which arrested the cell cycle in the G1 phase and induced apoptosis [65]. As shown in Scheme 2, a novel class of dehydroabietic acid acyl-thiourea derivatives (**29**(**a**-**o**)−**30**(**a**-**o**)) were obtained and their antitumor activity was evaluated against HeLa, SK-OV-3, HL-7702, and MGC-803 cell lines. Most of these compounds demonstrated cytotoxicity, especially compound **30n** (IC_50_: 6.58 ± 1.11 μM against HeLa) exhibited better inhibitory effect than 5-Fu (a commercial antitumor drug, IC_50_: 36.58 ± 1.55 μM). Moreover, compound **30n** could block HeLa cells in the S phase and induce HeLa cell apoptosis via the mitochondrial pathway [66]. In 2020, Li et al. designed and synthesized compounds **31** and **32** (Scheme 2). Compared with cisplatin and oxaliplatin, compound **31** had better cytotoxic activity on A431 cells and a stronger ability to bind with DNA [67]. Moreover, compounds **33a**−**33m** (Scheme 2) were synthesized as antitumor and antimicrobial agents. Compound **33e**, a MIC value of 1.9 μg/mL, demonstrated the most potent inhibitory activity against *B. subtilis*. Meanwhile, compound **33m** showed anticancer activity comparable to the positive control etoposide [68].

**Scheme 3:**
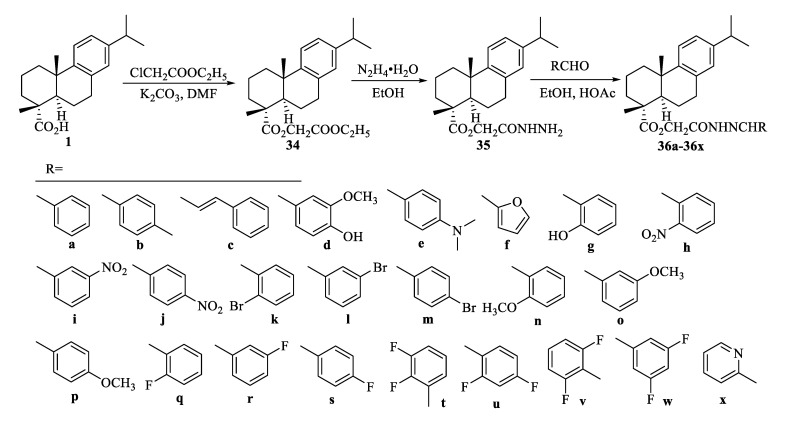
Synthesis of compounds **36a**−**36x**.

First, dehydroabietic acid (**1**) reacted with ethyl chloroacetate to obtain intermediate compound **34**. Aminolysis of **34** gave hydrazide **35**, which reacted with different aromatic aldehydes to obtain the target products **36a**−**36x** (Scheme 3). Compounds **36i**, **36k**, **36l**, **36w**, and **36x** displayed potent anticancer activity. Especially compound **36w** exhibited the promising inhibitory effects against HeLa and BEL-7402 cells with IC_50_ values of 2.21 and 14.46 µM, respectively [69].

**Scheme 4:**
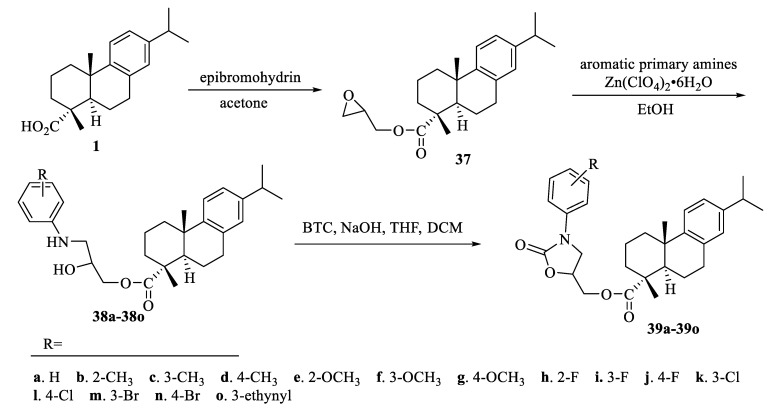
Synthesis of compounds **39a**−**39o**.

As described in Scheme 4, Wang et al. designed and synthesized a novel class of dehydroabietic acid derivatives containing oxazolidinone moiety (**39a**−**39o**). Compound **39j** not only induced apoptosis of MGC-803 cells (IC_50_ = 3.82 ± 0.18 µM), but also arrested the cell cycle in the G1 phase [70]. 

**Scheme 5:**
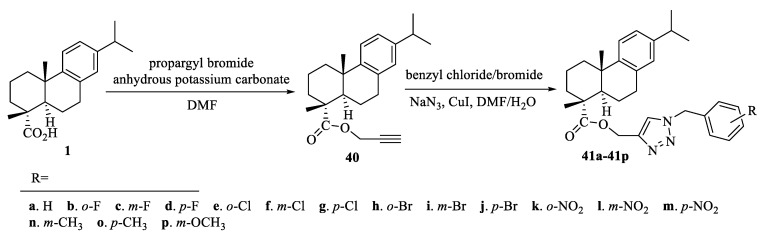
Synthesis of compounds **41a**−**41p**.

Additionally, dehydroabietic acid containing 1,2,3-triazole derivatives (**41a**−**41p,** Scheme 5) were prepared. The most potential compounds 41c (5.90 ± 0.41 µM) and 41k (6.25 ± 0.37 µM) exhibited better antiproliferation activity against HepG2 cells than that of cisplatin [71]. 

**Scheme 6:**
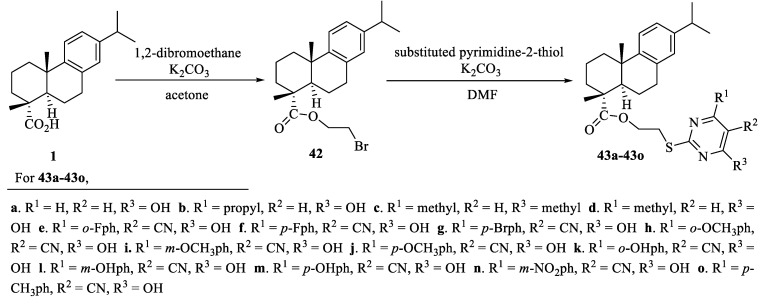
Synthesis of compounds **43a**−**43o**.

As depicted in Scheme 6, in the presence of anhydrous potassium carbonate, dehydroabietic acid (**1**) reacted with 1,2-dibromoethane to give intermediate **42**. Target compounds **43a**−**43o** were obtained by reacting compound **42** with substituted pyrimidine-2-thiol. Compound **43b** showed good inhibitory activity on the tested cells (IC_50_: 7.00–11.93 μM), but it had a little toxic effect on normal cells. The mechanism of action of compound **43b** was to block MCF-7 cells in the S phase and induced apoptosis [72].

**Scheme 7:**
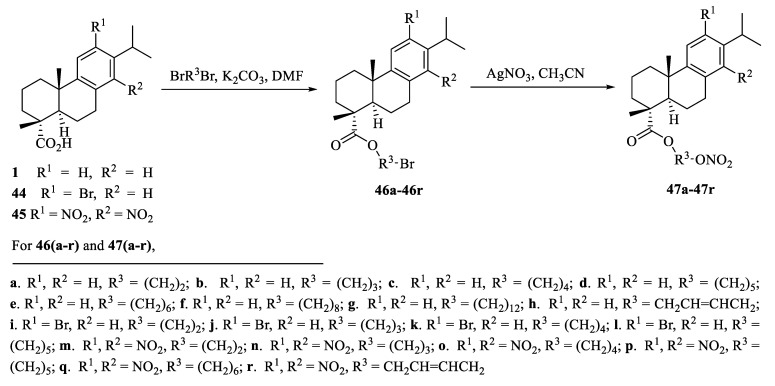
Synthesis of compounds **47a**−**47r**.

As described in Scheme 7, Li et al. synthesized a class of dehydroabietic acid-nitrate conjugates (**47a**−**47r**) as antitumor agents. Compound **47n** showed the most promising activity against the BEL-7402 cell line (IC_50_: 11.23 ± 0.21 μM). Compound **47j** exhibited the most potent cytotoxicity against the CNE-2 cell line (IC_50_: 8.36 ± 0.14 μM) and evaluation of NO release indicated that the cytotoxic activity improved with the increase of the amount of NO produced in the CNE-2 cell line [73].

**Scheme 8:**
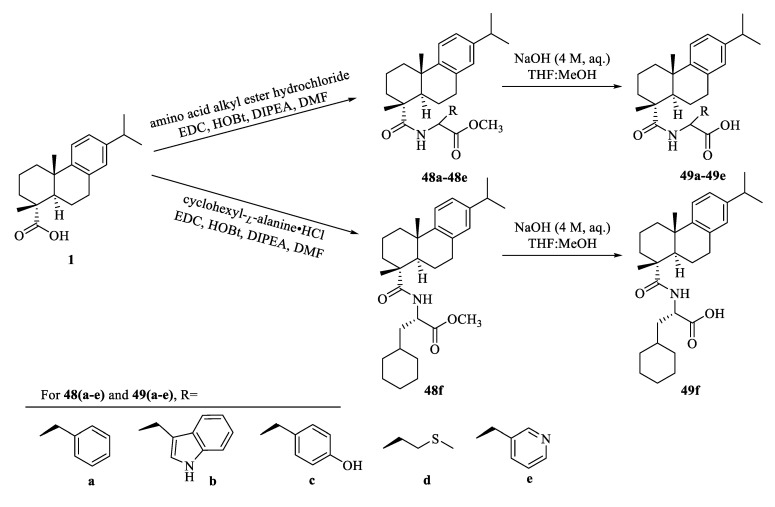
Synthesis of compounds **49a**−**49f**.

L-/D-amino acids and unusual amino acids were used as side chains to prepare new dehydroabietic acid derivatives (**49a**−**49f**, Scheme 8). Compounds **49b** and **49f** were the most effective anti-biofilm agents, which can quickly and effectively destroy membrane integrity [74].

**Scheme 9:**
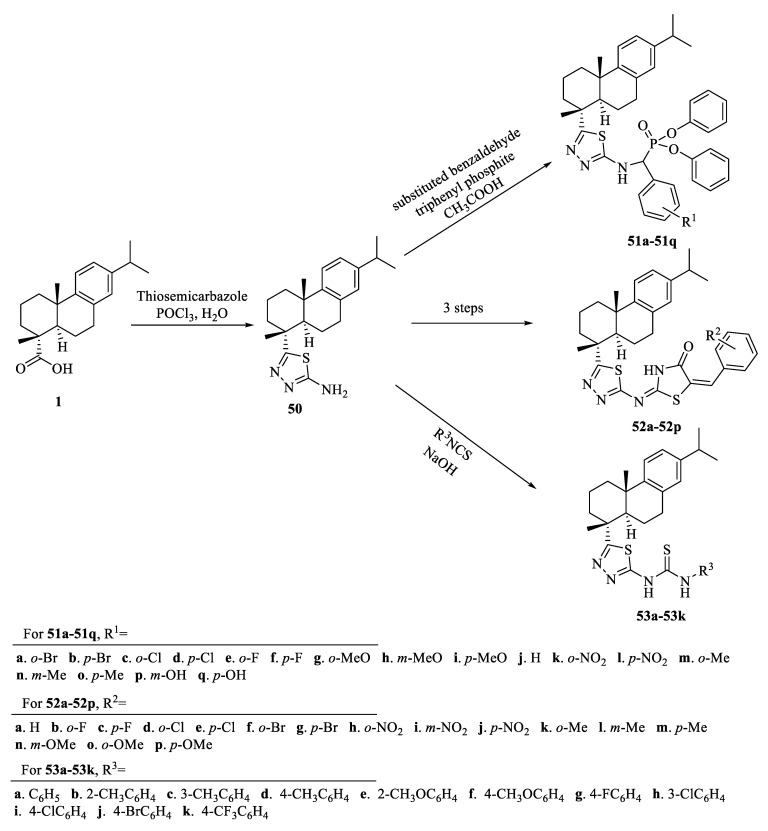
Synthesis of compounds **51a**−**51q, 52a**−**52p** and **53a**−**53k**.

As illustrated in Scheme 9, compounds **51a**−**51q** were smoothly synthesized by cyclization and Mannich-type reactions. At 50 µg/mL, compounds **51e**, **51f**, **51h**, and **51i** had better antifungal activity than azoxystrobin (a commercial antifungal drug) [75]. A series of dehydroabietic acid derivatives containing 1,3,4-thiadiazole-thiazolidinone (**52a**−**52p**) were synthesized as antifungal agents. They showed excellent antifungal activities against *Gibberella zeae* at a concentration of 50 µg/mL. Additionally, the antifungal activity of compounds **52c**, **52f**, and **52n** (inhibition rate: 91.3%) was comparable to that of azoxystrobin (positive control) [76]. Mo et al. designed and synthesized a series of novel dehydroabietic acid derivatives containing 1,3,4-thiadiazole thiourea (**53a**−**53k**) to determine their insecticidal activity against *Helicoverpa armigera*, *P. xylostella,* and *Ostrinia mubilalis*. Compounds **53a** and **53c** showed excellent insecticidal activity against *H. armigera* at 200 mg/L with mortality rates of 93.3% and 83.3%, respectively. The control effects of compounds **53b**, **53c**, **53d**, and **53i** against corn borer were 66.7%, 66.7%, 73.3%, and 60%, respectively [77].

### 3.2. Structural Modification at B Ring of Dehydroabietic Acid

**Scheme 10:**
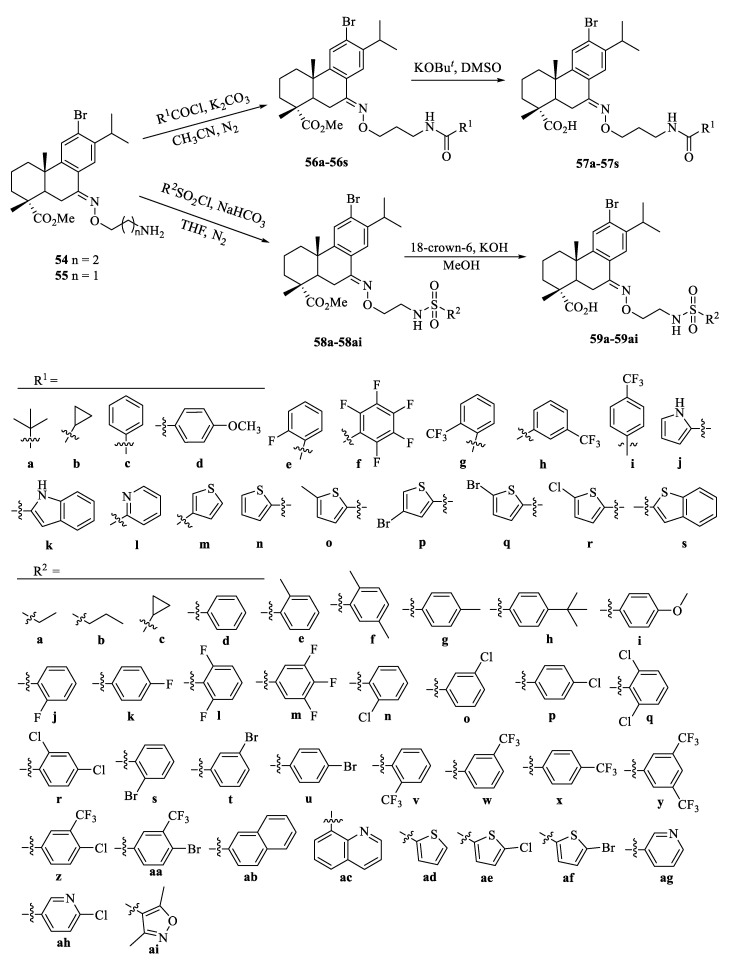
Synthesis of compounds **57a**−**57s** and **59a**−**59ai**.

A series of 7-*N*-acylaminopropyloxime derivatives of dehydroabietic acid (**57a**−**57s**, Scheme 10) were synthesized, and their antibacterial activities against *S. aureus* Newman strain, NRS-1, NRS-70, NRS-100, NRS-108, and NRS-271 were studied. Compound **57j** showed high antibacterial activities against five kinds of multi-drug resistant *S. aureus* with MIC values of 1.56–3.13 μg/mL [78].

In 2018, Zhang et al. synthesized a series of *N*-sulfonaminoethyloxime derivatives of dehydroabietic acid from compound **55** (Scheme 10). The MIC value of analog **59w** against *S. aureus* Newman was 0.39–0.78 μg/mL**,** exhibiting the highest activity. In addition, the remaining analogs also showed better antibacterial activities against five multi-drug resistant *S. aureus*, with MIC values ranging from 0.78 to 1.56 μg/mL [79].

**Scheme 11:**
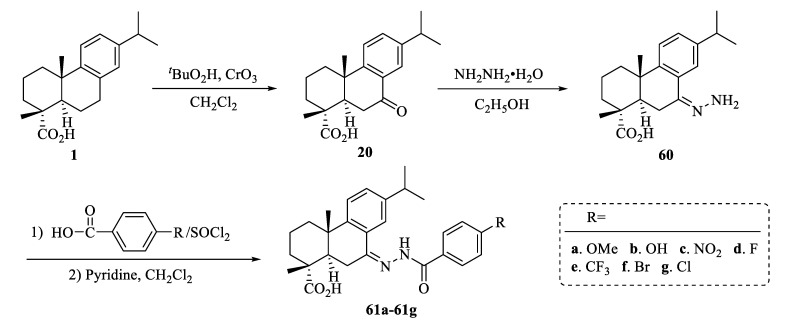
Synthesis of compounds **61a**−**61g**.

A series of hydrazone derivatives of dehydroabietic acid (**61a**−**61g**, Scheme 11) were synthesized. All compounds showed antibacterial activities against *Escherichia coli*, *S. aureus*, and *B. subtilis*. Especially compound **61d** was the potent antimicrobial agent against *B. subtilis* and *S. aureus* [80].

**Scheme 12:**
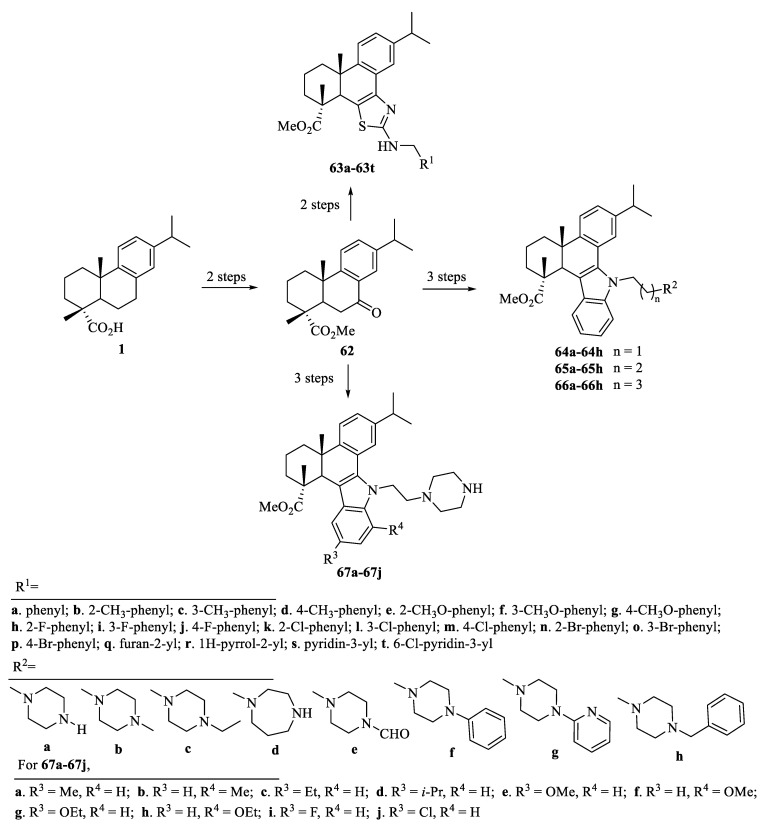
Synthesis of compounds **63a**−**63t, 64**(**a**-**h**)−**66(a**-**h)** and **67a**−**67j**.

Chen et al. synthesized a series of dehydroabietic acid derivatives (**63a**−**63t**, Scheme 12) to find novel and effective antitumor agents, and tested *in vitro* cytotoxic activity against HepG2, SCC9, and 293T by CCK-8 method. Compounds **63r** and **63s** had a certain cytotoxic activity to cancer cells, but were weak to normal cells. Two compounds could inhibit the PI3K/AKT/mTOR signaling pathway to exert anti-cancer effects. The results of molecular docking studies indicated that the compounds may inhibit the pathway through ATP competition [81].

As depicted in Scheme 12, compounds **64(a**-**h)**−**66(a**-**h)** and **67a**−**67j** were synthesized smoothly from dehydroabietic acid. Among these compounds, compound **67g** had excellent anti-proliferative activity against three liver cancer cell lines (SMMC-7721, HepG2, and Hep3B), with IC_50_ values of 0.51–1.39 μM. In addition, compound **67g** may inhibit MEK1 kinase activity, increase intracellular ROS levels, destroy cell membranes, and thus cause HepG2 cell apoptosis [82].

### 3.3. Structural Modification at C Ring of Dehydroabietic Acid

**Scheme 13:**
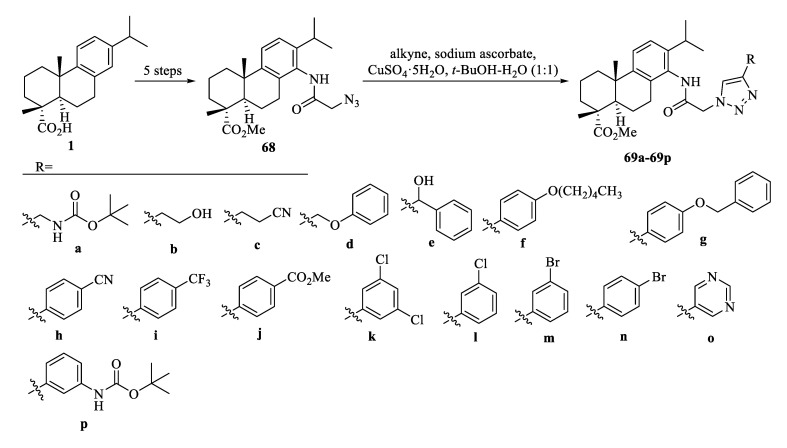
Synthesis of compounds **69a**−**69p**.

In order to find effective new antimicrobial agents, a class of new derivatives of dehydroabietic acid (**69a**−**69o**, Scheme 13) was obtained. Compound **69o** showed excellent antibacterial activity against both Gram-negative and positive bacteria (MIC: 1.6–3.1 μg/mL) but had no obvious toxicity to mammalian cells. In addition, compound **69o** (containing 1,2,3-triazole moiety at the C-14 position) had good drug-like properties [83]. Additionally, compound **69p** (IC_50_ values from 0.7 to 1.2 μM) not only induced apoptosis of MDA-MB-231 cells but also had weak toxicity to normal cells. And its anti-proliferative activity was better than 5-Fu (average IC_50_: 16.1 μM) [84].

**Scheme 14:**
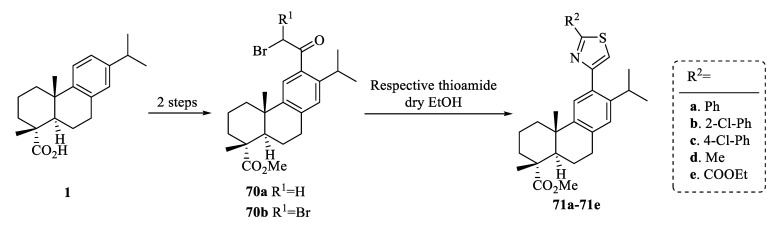
Synthesis of compounds **71a**−**71e**.

Several derivatives of dehydroabietic acid containing 12-thiazole moiety (**71a**−**71e**) were designed and synthesized as shown in Scheme 14. In order to understand the importance of hABHD16A inhibition *in vivo*, the feasibility of these derivatives as a starting point for the design of selective ABHD16A (a new target for inflammation-mediated pain) inhibitors was investigated. Compound **71d** had an IC_50_ value of 3.4 ± 0.2 µM with good selectivity [85].

**Scheme 15:**
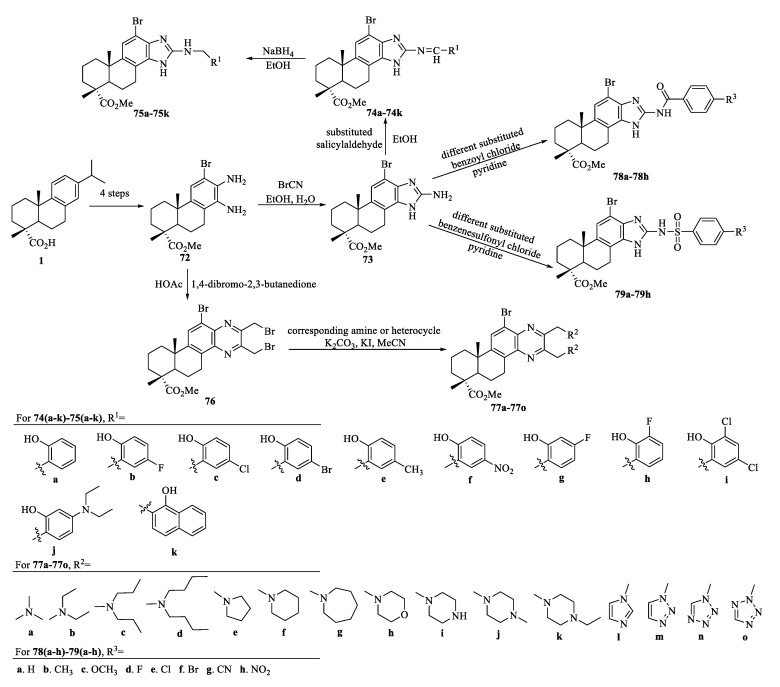
Synthesis of compounds **74**(**a-k**)−**75(a-k**)**, 77a**−**77o and 78**(**a-h**)−**79(a-h**).

Gu et al. studied the cytotoxicity of a series of newly synthesized dehydroabietic acid derivatives (**74**(**a**-**k**)−**75**(**a-k**), Scheme 15) on liver cancer cells and found that most of the compounds had obvious cytotoxic activity on SMMC-7721 and HepG2 liver cancer cells with low toxicity on normal human liver cells. Among them, compounds **74b** and **74e** showed the best cytotoxicity against SMMC-7721 and HepG2 cells (IC_50_ values: 0.36 ± 0.13 and 0.12 ± 0.03 μM, respectively). In addition, compound **74b** not only caused SMMC-7721 cells’ cell cycle arrest in the G2/M phase, but also induced apoptosis [86]. 

Subsequently, novel quinoxaline derivatives of dehydroabietic acid (**77a**−**77o**, Scheme 15) were synthesized and tested against MCF-7, SMMC-7721, and HeLa cancer cell lines. Compound **77b** caused SMMC-7721 cells to arrest in the G0/G1 phase of the cell cycle and induced their apoptosis in a dose-dependent manner. Its IC_50_ values against three different cancer cells were 0.72–1.78 μM, which had the best anti-cancer effect, while its toxicity to LO2 cells was reduced [87]. In 2020, a series of *N*-(1*H*-benzo[*d*]imidazol-2-yl)benzamide/benzenesulfonamide derivatives of dehydroabietic acid (**78**(**a**-**h**)−**79**(**a**-**h**), Scheme 15) were prepared. Compounds **78a**, **78g**, **78h**, **79g,** and **79h** showed good anticancer effects on at least one cancer cell line (IC_50_ <10 μM), while compounds **1**, **78b**, **78c**, **78e,** and **78f** were inactive (IC_50_ > 50 μM). In addition, benzenesulfonamide derivatives (**79a**−**79h**) had stronger inhibitory activity than benzamide ones (**78a**−**78h**). The most cytotoxic compound **79h** (IC_50_: 0.87–9.39 μM) can arrest MCF-7 cells in the S phase through ROS-mediated mitochondrial pathway, and finally induce MCF-7 cell apoptosis [88].

**Scheme 16:**
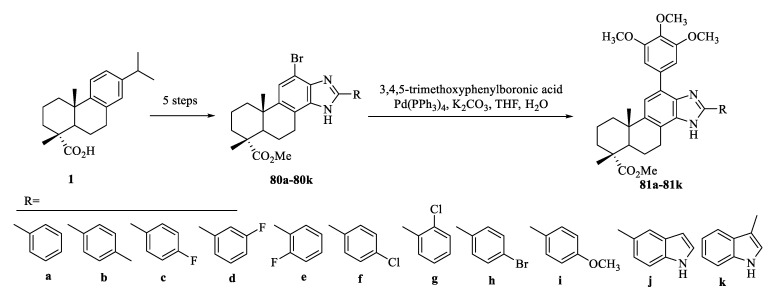
Synthesis of compounds **80(a-k)**−**81(a-k)**.

Miao et al. synthesized a series of novel 2-aryl-benzimidazole derivatives of dehydroabietic acid (**80**(**a**-**k**)−**81**(**a**-**k**), Scheme 16). Compound **80j** exhibited the strongest cytotoxic activity with the IC_50_ value of 0.08–0.42 μM. In addition, further in-depth exploration elucidated that compound **80j** significantly inhibited the migration ability of SMMC-7721 cells, and induced cell cycle arrest and apoptosis of the cells in the G2/M phase. Through tubulin polymerization and immunofluorescence assays, it was found that compound **80j** not only inhibited tubulin polymerization but also destroyed the intracellular microtubule network [89].

**Scheme 17:**
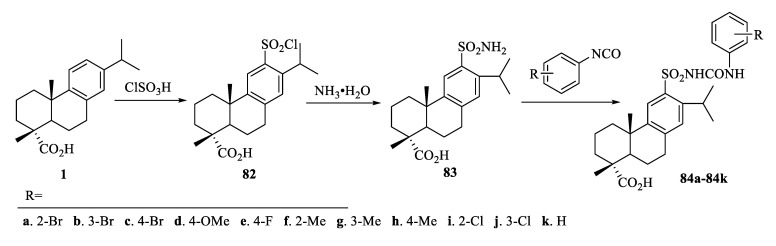
Synthesis of compounds **84a**−**84k**.

As shown in Scheme 17, a series of sulfonylurea derivatives of dehydroabietic acid (**84a**−**84k**) were prepared. Compounds **84a**, **84c**, **84e**, and **84i** had higher anti-HCT-116 cell activity than 5-Fu. In particular, compound **84a**, an IC_50_ value of which was 1.18 ± 0.52 μM, showed the best anti-tumor proliferation activity [90].

Some IC_50_ values and MIC values of the most active derivatives were listed in Table 2 and Table 3.

### 3.4. Biotransformation of Dehydroabietic Acid

**Scheme 18:**
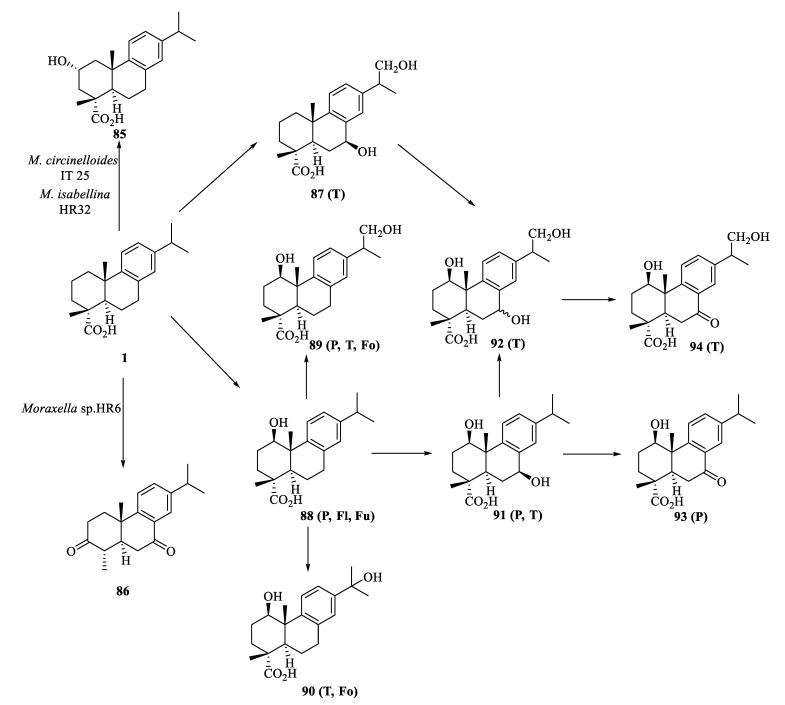
Possible pathways for the biotransformation of dehydroabietic acid by Mucor circinelloides IT25, Mortierella isabellina HR32, *Moraxella* sp. HR6, Trametes versicolor (**T**), Phlebiopsis gigantea (**P**), Flavobacterium resinovorum (**Fl**), Fusarium oxyosporum/F. moniliforme (**Fu**) and Fomes annosum (**Fo**).

Possible pathways for the biotransformation of dehydroabietic acid by Mucor circinelloides IT25, Mortierella isabellina HR32, Moraxella sp. HR6, Trametes versicolor, Phlebiopsis gigantea, Flavobacterium resinovorum, Fusarium oxyosporum/F. moniliforme and Fomes annosum were shown in Scheme 18. These microorganisms were some of the previously reported microorganisms with hydroxylated metabolites. Two molds (M. circinelloides IT 25 and M. isabellina HR32) regioselectively and stereoselectively hydroxylated dehydroabietic acid to 2α-hydroxydehydroabietic acid (**85**). Dehydroabietic acid was oxidized at the C-3 and C-7 positions, decarboxylated at the C-4 position by the action of bacteria Moraxella sp. HR6, and finally converted to 3,7-dioxodehydroabietin (**86**) [91]. The first step in the degradation of dehydroabietic acid was caused by two fungi, T. versicolor and P. gigantea, and a stereoselective hydroxylation was at the C-1 position, followed by further hydroxylation at the C-7 or C-16 position to form dihydroxylated compounds **89** and **91**. Compound **87** or **91** was further hydroxylated at the C-1 or C-7 position, resulting in trihydroxylated compound **92**. The hydroxyl group at the C-7 position of compounds **91** and **92** can be further oxidized to a carbonyl functional group to afford compounds **93** and **94** [92,93].

## 4. Total Synthesis of Dehydroabietic Acid

Dehydroabietic acid has attracted the attention of researchers because of its diverse biological activities. However, separation and purification of dehydroabietic acid usually required expensive catalysts, and the organic amine salt method was more toxic and polluted the environment [10]. Therefore, it was particularly important to find other methods for synthesis of dehydroabietic acid. Some total synthesis processes of dehydroabietic acid were as follows.

**Scheme 19:**
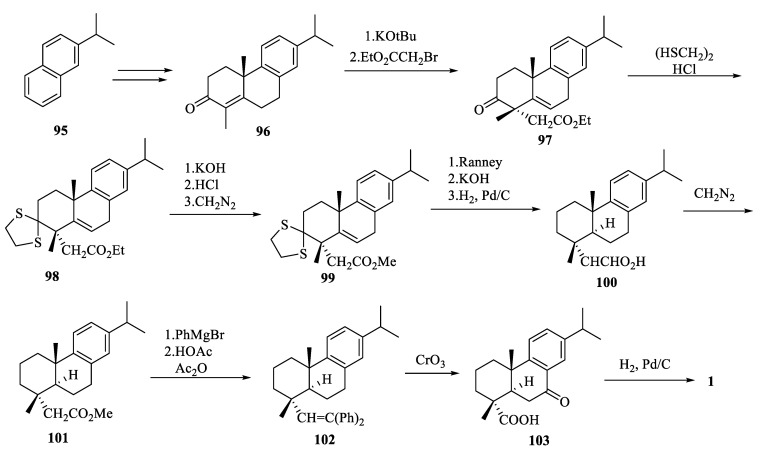
Total synthesis of dehydroabietic acid from 2-isopropylnaphthalene.

In 1956, the first synthesis of dehydroabietic acid was begun with 2-isopropylnaphthalene (**95**) as the starting material (Scheme 19). The most important step was to define the C-4 center, that is, to alkylate phenanthrone (**96**) with ethyl bromoacetate. The enone (**96**) was then alkylated to obtain the keto ester (**97**), which reacted with HSCH_2_CH_2_SH to form the thioketal (**98**). The thioketal (**98**) was converted into its methyl ester (**99**) by ester hydrolysis and diazotization reactions. After Raney nickel desulfurization, ester hydrolysis, and Pd/C hydrogenation, intermediate **100** was obtained, which reacted with CH_2_N_2_ to produce methyl ester compound **101**. Compound **103** was obtained by Barbiere Wieland degradation of **101**. Finally, dehydroabietic acid (**1**) was obtained by hydrogenation of **103** on Pd/C [94].

**Scheme 20:**
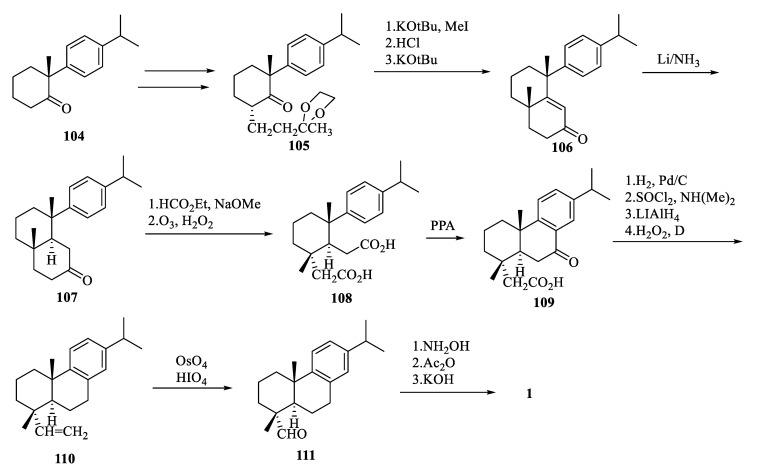
Total synthesis of dehydroabietic acid from 2-methyl-2-(p-isopropylphenyl)cyclohexanone.

The second method started with 2-methyl-2-(p-isopropylphenyl)cyclohexanone (**104**) was described in Scheme 20. The key steps were stereoselective alkylation of **105** with KO*t*-Bu and MeI, and reduction with Li in liquid ammonia to give decalone **107**, which reacted with HCO_2_Et, O_3_, and polyphosphoric acid (PPA) to give tricyclic keto acid **109**. After the hydrogenolysis reaction, the amine oxide pyrolyzed to afford the vinylic compound **110**, which reacted with OsO_4_ and HIO_4_ to give the corresponding aldehyde (**111**). Finally, dehydroabietic acid (**1**) was obtained by reaction of 111 with NH_2_OH and KOH [94].

**Scheme 21:**
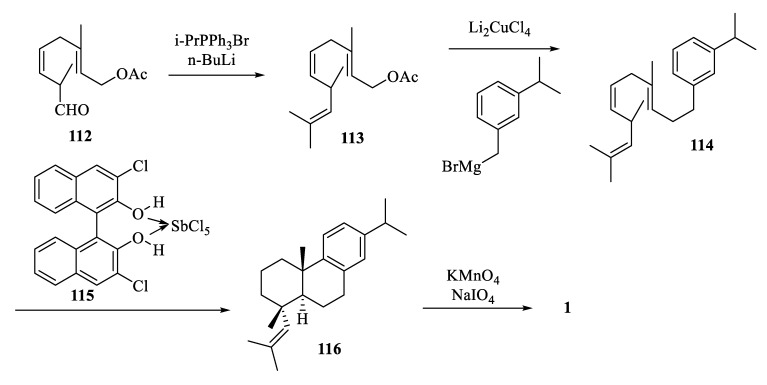
Total synthesis of dehydroabietic acid from the geranyl acetate derivative.

The third method was a short enantioselective synthesis starting from the geranyl acetate derivative (**112**) as shown in Scheme 21. Compound **112** was converted to alkene **113** by the Wittig reaction, which was further catalyzed by Li_2_CuCl_4_ to give compound **114**. The enantioselective cyclization of **114** and **115**/SbCl_5_ complex afforded compound **116,** which was a key step in the reaction. Finally, compound **116** reacted with KMnO_4_ and NaIO_4_ to obtain dehydroabietic acid (**1**) [94].

## 5. Structure-Activity Relationships of Dehydroabietic Acid and Its Derivatives

**Figure 6:**
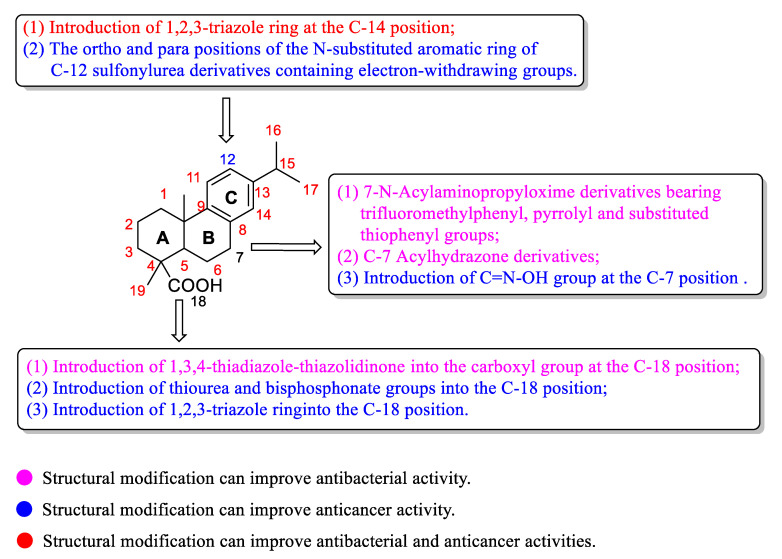
The overall SARs of dehydroabietic acid and its derivatives for antibacterial and anticancer activities.

The SARs analysis of dehydroabietic acid and its derivatives about the antibacterial and anticancer activities (Figure 6) were as follows: 

(1) For antibacterial activity: (a) Introduction of trifluoromethyl phenyl, pyrrolyl, or substituted thiophenyl groups (**57h**−**57j** and **57p**−**57s**) into the oxime derivatives at the C-7 position of dehydroabietic acid was beneficial to the antibacterial activity [78]. C-7 Acylhydrazone derivatives of dehydroabietic acid (especially *p*-fluorobenzoyl hydrazone) can increase the antibacterial activity [80]. (b) Introduction of 1,3,4-thiadiazole-thiazolidinone into the C-18 carboxyl group of dehydroabietic acid can effectively improve the antibacterial activity [76]. (c) Introduction of 1,2,3-triazole ring at the C-14 position of dehydroabietic acid can improve the antibacterial activity [83].

(2) For anticancer activity: (a) Introduction of the C=N-OH group at the C-7 position can improve anti-tumor activity [64]. (b) Introduction of thiourea and bisphosphonate groups into the C-18 position of dehydroabietic acid was beneficial to the anticancer activity [65]. (c) 1,2,3-Triazole ring at the C-18 position played a key role for dehydroabietic acid derivatives showing the anti-cancer effect [71,84]. (d) C-12 sulfonylurea derivatives of dehydroabietic acid bearing electron-withdrawing groups at the ortho or para position on the N-substituted aromatic ring, can improve the antiproliferative activity [90]. 

Researchers expected to synthesize high bioactive compounds with low toxicity. Herein, we summarized some IC_50_ values of the most active derivatives against human normal cell lines (Table 4). Most of these derivatives exhibited more potent activity than dehydroabietic acid against cancer cell lines, and they showed low cytotoxicity against human normal cells. The IC_50_ value of compound **36w** against HeLa cells (2.21 ± 0.04 μM) was about 17-folds higher than that of the precursor (37.40 ± 0.64 μM), while the cytotoxicity of compound **36w** against normal HL-7702 human liver cell was lower (66.08 ± 1.84 μM) [69]. Compound **39j** exhibited good cytotoxicity against the tested cancer cell lines (IC_50_ values:.82–17.76 μM) compared to the lead compound, with no increase in cytotoxicity against normal cells (IC_50_ > 100 μM) [70]. In addition, the anticancer activity of compounds **41c**, **41k**, **47j,** and **47n** was significantly improved, but their toxicity against human normal cells was not increased compared to that of dehydroabietic acid; that is, they exhibited excellent selectivity between normal and cancer cells [71,73]. The cytotoxicity of compound **77b** against normal human liver cell LO2 (IC_50_: 11.09 ± 0.57 µM) was much lower than that of compound **77b** against MCF-7, SMMC-7721, and HeLa cancer cells (IC_50_ values: 0.72–1.78 µM). However, compared with that of dehydroabietic acid (IC_50_ > 50 μM), it was more toxic to normal cells [87]. Compound **79h** was also more cytotoxic against normal human LO2 cell (IC_50_: 42.83 ± 3.18 μM) than dehydroabietic acid (IC_50_ > 50 μM) [88]. Based on the above results, when compared with the precursor, the bioactivity of some compounds was significantly improved after structural modifications, but their toxicity against normal human cells was also increased. Fortunately, compounds **39j**, **41c**, **41k**, **47j,** and **47n** not only showed promising biological activity, but also had good selectivity between normal and malignant cells; so they can be further studied and developed as potential anticancer drugs.

## 6. Conclusions

Dehydroabietic acid, a tricyclic diterpenoid resin acid, showed a wide range of biological activities. The structural characteristics suggested that dehydroabietic acid had multiple structural modification sites, and using it as a lead compound can improve its application value. This review gave an overview of the biological activities, structural modifications, biotransformation, total synthesis, and structure-activity relationships of dehydroabietic acid and its derivatives. Although several structural modifications of dehydroabietic acid and its derivatives were summarized, their properties were mainly focused on anticancer and antibacterial activities. Obviously, their agricultural and other activities needed to be expanded in the future. Therefore, we hope that this review can provide references for further research and development of dehydroabietic acid and its derivatives, as well as their applications as drugs and pesticides in the medical and agricultural fields.

## Figures and Tables

**Table 1:** Some MIC values of dehydroabietic acid derivatives against different bacteria.

Compound	Bacteria	MIC Value
**5**	*B. subtilis*	4 μg/mL [36]
**5**	*S. aureus*	2 μg/mL [36]
**7**	methicillin resistant *S. aureus*	32 μg/mL [39]
**8**	LA-MRSA LGA251(ST425-XI)	3.9 μg/mL [40]
**9**	*S. aureus* Newman	0.39–0.78 μg/mL [41]
**10**	NRS-70	1.25–1.56 μg/mL [41]
**11**	*S. aureus* Newman, NRS-1, NRS-70, NRS-100, NRS-108, and NRS-271	1.56–3.13 μg/mL [41]
**12**	*S. aureus* Newman, NRS-70, NRS-108, and NRS-271	1.56–3.13 μg/mL [41]

**Table 2:** Some IC_50_ values of the most active derivatives against different cancer cell lines.

Compound	Cancer Cell Line	IC_50_ Value
**22f**	HeLa	7.76 ± 0.98 μM [64]
**28e**	SK-OV-3	1.79 ± 0.43 μM [65]
**30n**	HeLa	6.58 ± 1.11 μM [66]
**36w**	HeLa	2.21 ± 0.04 μM [69]
**36w**	BEL-7402	14.46 ± 0.22 μM [69]
**39j**	MGC-803	3.82 ± 0.18 μM [70]
**41c**	HepG2	5.90 ± 0.41 μM [71]
**41k**	HepG2	6.25 ± 0.37μM [71]
**43b**	MCF-7	7.00 ± 0.96 μM [72]
**47n**	BEL-7402	11.23 ± 0.21 μM [73]
**47j**	CNE-2	8.36 ± 0.14 μM [73]
**63r**	HepG2	24.41 ± 0.26 μM [81]
**63s**	HepG2	22.92 ± 0.24 μM [81]
**67g**	SMMC-7721	1.39 ± 0.13 μM [82]
**67g**	HepG2	0.51 ± 0.09 μM [82]
**67g**	Hep3B	0.73 ± 0.08 μM [82]
**69p**	MDA-MB-231	0.7 ± 0.1 μM [84]
**74b**	SMMC-7721	0.36 ± 0.13 μM [86]
**74e**	HepG2	0.12 ± 0.03 μM [86]
**77b**	SMMC-7721	0.72 ± 0.09 μM [87]
**79h**	MCF-7	0.87 ± 0.18 μM [88]
**80j**	SMMC-7721	0.08 ± 0.01 μM [89]
**84a**	HCT-116	1.18 ± 0.52 μM [90]

**Table 3:** Some MIC values of the most active compounds against different bacteria.

Compound	Bacteria	MIC Value
**33e**	*B. subtilis*	1.9 μg/mL [68]
**57j**	*S. aureus* NRS-70, NRS-100, NRS-108 and NRS-271	1.56–2.5 μg/mL [78]
**69o**	*S. aureus*, *E. coli* and *P. fluorescens*	1.6 μg/mL [83]

**Table 4:** Some IC_50_ values of the most active derivatives against human normal cell lines.

Compound	Human Normal Cell Line	IC_50_ Value
**22f**	HL-7702	53.78 ± 1.4 μM [64]
**36w**	HL-7702	66.08 ± 1.84 μM [69]
**39j**	LO2	>100 μM [70]
**41c, 41k**	HL-7702	>100 μM [71]
**47j**	HL-7702, NP69	>100 μM [73]
**47n**	NP69	>100 μM [73]
**47n**	HL-7702	88.18± 0.23 μM [73]
**67g**	QSG-7701	12.52 ± 0.58 μM [80]
**74b**	LO2	3.89 ± 0.29 μM [86]
**74e**	LO2	3.02 ± 0.21 μM [86]
**77b**	LO2	11.09 ± 0.57 μM [87]
**79h**	LO2	42.83 ± 3.18 μM [88]
**80j**	QSG-7701	5.82 ± 0.38 μM [89]

## Abbreviations

AP-1	Activating protein-1
5-Fu	5-Fluorouracil
G6Pase	Glucose-6-Phosphatase
GS	Glycogen Synthase
IC_50_	Half-inhibitory concentration
MCP-1	Monocyte chemoattractant protein-1
MIC	Minimum inhibitory concentration
NF-κB	Nuclear factor-κB
Nrf2	Nuclear factor erythroid 2-related factor 2
NO	Nitric oxide
PARP	Poly (ADP-ribose) polymerase
PPA	Polyphosphoric acid
PPAR	Peroxisome proliferator-activated receptors
ROS	Reactive oxygen species
TAK1	Transforming growth factor β-activated kinase 1
TNF-α	Tumor necrosis factor-α
